# Inadequate tissue mineralization promotes cancer cell attachment

**DOI:** 10.1371/journal.pone.0237116

**Published:** 2020-08-28

**Authors:** Ediz Sariisik, Domenik Zistl, Denitsa Docheva, Arndt F. Schilling, Martin Benoit, Stefanie Sudhop, Hauke Clausen-Schaumann

**Affiliations:** 1 Center for Applied Tissue Engineering and Regenerative Medicine (CANTER), Munich University of Applied Sciences, Munich, Germany; 2 Chair of Applied Physics, Ludwig-Maximilians-Universität, Munich, Germany; 3 Center for NanoScience, Ludwig-Maximilians-Universität, Munich, Germany; 4 Department of Trauma Surgery, Experimental Trauma Surgery, University Regensburg Medical Centre, Regensburg, Germany; 5 Clinic for Trauma Surgery, Orthopaedics, and Plastic Surgery, University Medical Center Göttingen, Göttingen, Germany; LAAS-CNRS, FRANCE

## Abstract

Bone metastases are a frequent complication in prostate cancer, and several studies have shown that vitamin D deficiency promotes bone metastases. However, while many studies focus on vitamin D’s role in cell metabolism, the effect of chronically low vitamin D levels on bone tissue, i.e. insufficient mineralization of the tissue, has largely been ignored. To investigate, whether poor tissue mineralization promotes cancer cell attachment, we used a fluorescence based adhesion assay and single cell force spectroscopy to quantify the adhesion of two prostate cancer cell lines to well-mineralized and demineralized dentin, serving as biomimetic bone model system. Adhesion rates of bone metastases-derived PC3 cells increased significantly on demineralized dentin. Additionally, on mineralized dentin, PC3 cells adhered mainly via membrane anchored surface receptors, while on demineralized dentin, they adhered via cytoskeleton-anchored transmembrane receptors, pointing to an interaction via exposed collagen fibrils. The adhesion rate of lymph node derived LNCaP cells on the other hand is significantly lower than that of PC3 and not predominately mediated by cytoskeleton-linked receptors. This indicates that poor tissue mineralization facilitates the adhesion of invasive cancer cells by the exposure of collagen and emphasizes the disease modifying effect of sufficient vitamin D for cancer patients.

## Introduction

Bone metastases formation is a feared complication during cancer progression, because it is usually associated with poor prognosis for the patient. Such bone metastases frequently occur in prostate cancer as well as in other common cancer types [1–3]. Despite intensive efforts in the investigation of cancer spreading to and growth in the bone, the molecular mechanisms that initiate the adhesion of a tumor cell to bone and, thus, trigger the cancer cell colonialization, are still not completely understood. Nevertheless, the complex and bidirectional interplay of cancer cells with proteins from the bone extracellular matrix and bone cells is supposed to be a key factor for bone invasion and metastasis formation [4–7]. By mediating the initial contact of tumor cells to the bone extracellular matrix (ECM), adhesion molecules, such as integrin receptors, together with ECM proteins, like collagen, may contribute to the invasive capability of cancer cells and their progression into the bone [8, 9]. In two previous studies, we have shown that the bone invasive prostate cancer cell line PC3 strongly interacts with collagen I, while the lymph node derived prostate cancer cell line LNCaP shows a much weaker interaction [10, 11]. At the same time, there is ample evidence that high vitamin D levels correlate positively with patient prognosis, and that vitamin D treatment prolongs disease-free intervals and increases survival times of the patients [12, 13]. Recent studies investigating the effect of vitamin D treatment in cancer therapy focus on the molecular response of cancer cells to increased vitamin D levels [14–16]. One prominent effect of chronically low vitamin D levels is a poor mineralization of mineralized tissues like bone and teeth [17, 18], exposing higher amounts of ECM proteins, such as collagen, at their surface [19]. The interplay between tissue mineralization and the initial interaction of circulating cancer cells with the mineralized surface has not yet been investigated.

Cell adhesion assays on mineralized and demineralized bone surfaces might address the question, if poor bone mineralization facilitates cancer cell attachment and thus possibly contributes to cancer cell spreading into the bone. However, for quantitative cell adhesion assays, flat substrates are required, and therefore bone samples with their trabecular structure and osteocyte lacunae are difficult to use. For the in vitro evaluation of bone resorption and osteoidosis, this problem is successfully solved by using elephant tusk dentin as a model system for bone [20–22]. Dentin is well suited as bone model system because both composite materials mainly consist of hydroxyapatite crystals and collagen I fibrils in similar proportions as major components [23]. Additionally, the ultrastructure of dentin as it was described by Jantou-Morris et al. [23, 24] closely resembles that of bone [25, 26]. In the present study, we have therefore chosen mineralized and acid treated (and thereby de-mineralized) elephant dentin as biomimetic model surfaces, mimicking the intact and the de-mineralized bone [22]. We have investigated the adhesive capacity of the two prostate carcinoma cell lines PC3 (bone marrow specific) and LNCaP (lymph node specific) to these model surfaces, using a fluorescence microscopy based cell adhesion assay in combination with atomic force microscopy (AFM) based single cell force spectroscopy (SCFS) [27]. To find out whether exposed collagen fibrils are indeed responsible for the observed increase in cell adhesion on acid treated substrates, we also used acid treated dentin substrates, where the exposed collagen fibrils were removed with collagenase, after the acid treatment.

## Materials and methods

### Sample preparation

Tusk dentin from African elephant was seized by German customs authorities and kindly provided by the German federal office for nature conservation (Bundesamt für Naturschutz), Konstantinstrasse 110, 53179 Bonn, in accordance with the international laws of species protection. Dentin bars were cut into small chips of 10 x10 x 1 mm in size using a diamond saw (PSI Grunewald GmbH & Co. KG, Laudenbach, Germany). In order to remove saw marks and to obtain a smooth surface, all dentin chips were polished using a Rotopol-35 (Struers GmbH, Willich, Germany) polisher with diamond pastes (Buehler GmbH, Dusseldorf, Germany) down to 1 μm particle size. Distilled water was used to wash off the particles of the previous polishing step.

For demineralization, the dentin chips were treated with 1 M hydrochloric acid (Carl Roth, Karlsruhe, Germany) for 5 minutes and then rinsed thoroughly with distilled water. For the removal of surface collagen, acid treated dentin chips were incubated in collagenase solution (11 mg/ml collagenase type II (Worthington, USA) dissolved in tricine-buffer: 33 mM tricine, 8 mM calciumchloride, 267 mM sodiumchloride, pH 7,5) overnight (9 to 12 hours) at room temperature and then rinsed with distilled water for 1 min prior to the experiments. As a control surface and for cell capture in AFM force spectroscopy experiments, we used cover slips coated with BSA (0.5% w/v, Sigma-Aldrich, Munich, Germany) at 4°C overnight as described in Sariisik et al. [10].

### Cell culture

PC3 (derived from bone metastatic site) and LNCaP (derived from lymph node metastases) cells were purchased from ATCC (Wesel, Germany). PC3 cells were cultivated using RPMI-1640 cell culture medium (Merck Biochrom, Berlin, Germany) supplemented with 10% FCS (Sigma-Aldrich, Munich, Germany) and 5% Penicillin-Streptomycin (Merck Biochrom, Berlin, Germany), LnCAP cells were maintained in MEM Alpha GlutaMAX culture media (Invitrogen, Karlsruhe, Germany) supplemented with 10% FCS and 5% Penicillin-Streptomycin as well. During routine cell culture, cells were grown to about 80% confluence at 37°C in 5% humidified CO_2_. Culture medium was changed three times a week, and both cell types were detached by treatment with 1% trypsin/EDTA solution (Merck Biochrom, Berlin, Germany) for splitting.

### Quantification of cell-dentin adhesion

In duplicates, 1x10^4^ prostate carcinoma (PC) cells were seeded in a volume of 100 μl cell culture medium (RPMI-1640 cell culture medium (Biochrom, Berlin, Germany) supplemented with 10% FCS (Sigma-Aldrich, Munich, Germany) and 5% Penicillin-Streptomycin (Biochrom, Berlin, Germany) for PC3 and MEM Alpha GlutaMAX culture media (Invitrogen, Karlsruhe, Germany) supplemented with 10% FCS and 5% Penicillin-Streptomycin for LnCAP) on untreated, acid treated and collagenase treated dentin chips, respectively. The cells were left to adhere to the dentin substrates for either 30 or 60 minutes under cell culture conditions and non-adherent cells were removed by gently washing the dentin chips with 37°C PBS (Biochrom, Berlin, Germany). Adherent cells were fixed with 4% (w/v) paraformaldehyde (Sigma-Aldrich, Munich, Germany) for 10 minutes at room temperature, followed by incubation with nuclear stain DAPI (Invitrogen, Karlsruhe, Germany) at a concentration of 1μg/ml for 5 minutes. For determination of cell count, micrographs of four randomly selected sections of each dentin chip with an area of 1.77x1.77mm^2^ were taken with an Axiocam MRm camera on an Axioscope 2 microscope (Carl Zeiss, Göttingen Germany) using a 20x objective. The number of adherent PC cells was evaluated by using the cell counter tool of the Image J software, version 1.40 (National Institute of Health, Bethesda, USA).

### AFM single cell force spectroscopy

Force Spectroscopy experiments were conducted using a NanoWizard II together with a CellHesion module (JPK Instuments, Berlin, Germany), mounted on a Zeiss Axiovert 200 M (Carl Zeiss, Goettingen, Germany) with a custom made temperature control unit. The set up was placed on an active isolation table (Micro 60, Halcyonics, Göttingen, Germany) and enclosed in a soundproof box. Tipless silicon nitride cantilevers with a nominal spring constant of 0.01 N/m (Tipless, MLCT-O10, Bruker, USA) coated overnight with 100 mg/ml Poly D-Lysine (PDL, Millipore, USA) were used for measurements. The spring constants of the force sensors were determined individually using the thermal noise method [28]. The determined spring constants varied between 0.012 and 0.017 N/m. PC cells were captured from a BSA-coated cover glass. Force-distance curves were recorded with the piezo travelling in a closed loop configuration up to 20 μm at an approach velocity of 7 μm/s until reaching a trigger force of 100pN and a retraction velocity of 3 μm/s. The contact time was set to 0 seconds, which results in an effective contact time of 0.2 ± 0.1 seconds. For details refer also to Sariisik et al. [10] and Reiprich et al. [29].

For all force spectroscopy experiments, untreated, acid treated and collagenase treated dentin chips were immobilized inside the same cell culture dish lid. Additionally, a cover slip coated with BSA (0.5% w/v) at 4°C overnight was placed adjacent to these substrates for cell capture. All substrates were washed with and covered by 1.5 ml fresh serum-free MEM Alpha medium (Invitrogen, Karlsruhe, Germany) supplemented with 15 mM HEPES (Sigma-Aldrich, Munich, Germany) resulting in a CO_2_-independent measurement medium with stable pH at 7.4 in ambient air. The culture dish lid was mounted on the temperature-controlled stage in the AFM and left to equilibrate in ambient air at 37°C for 10 minutes.

Cell capture and cell adhesion force measurements were performed as described above. Adhesion of each PC3 and LNCaP cell to all three dentin substrates (untreated, acid treated, collagenase treated) was investigated. To exclude biased results due to degradation effects of the cells or cells reacting on either substrate the order of the three substrates was varied stochastically within these experiments.

### Data analysis

Approximately 80–100 curves were collected from 3 differently treated dentin substrates for every cell in each experiment. In total, 8 PC3 and 8 LNCaP cells were measured on all three substrates. Force-distance curves were analyzed with a custom made force curve analysis algorithm, described elsewhere [30], to detect de-adhesion steps.

As described in detail in Sariisik et al. [10], we have calculated the following parameters from the obtained force-distance curves: The adhesion rate (percentage of force traces with adhesive steps to all force traces), the average number of steps (per force trace with adhesive steps), step positions (distance between the contact point and an adhesive step). We have also analyzed the force loading rates (slope of the force distance trace) prior to each step. Steps were defined as plateau steps (tethers) when the slope is 0 ± 10 pN/μm and jump steps (jumps) when the slope is < -10 pN/μm. For better visualization, the slope before each force step together with the distance from the surface at which the step occurs was evaluated and displayed in two-dimensional slope vs. step position density plots, refer to Sariisik et al., 2015 for details [31].

### Statistical analysis

We have used GraphPad Prism Version 8.4.3 software to perform the statistical analysis of the data.

**Fig 1:**
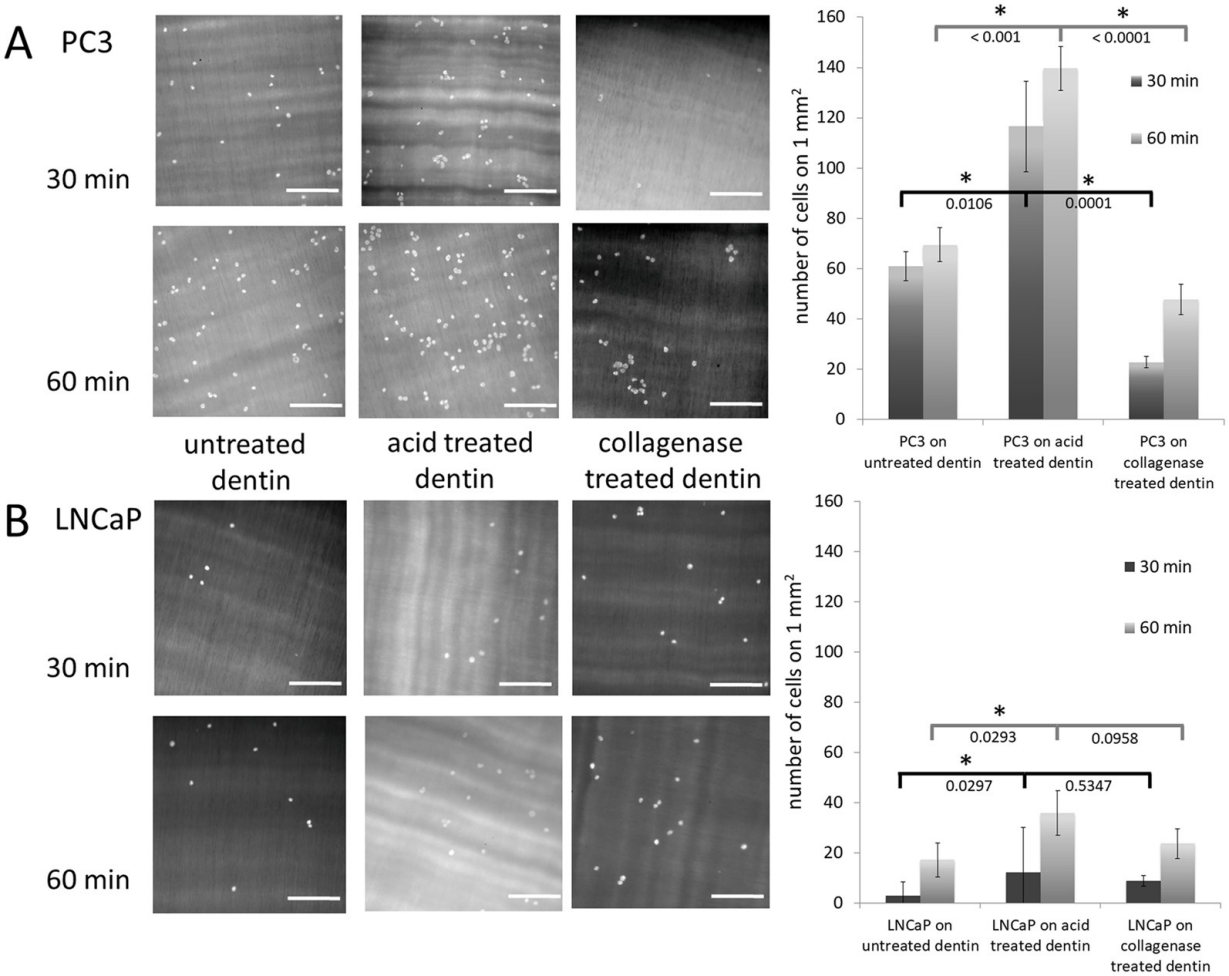
Cell adhesion of PC3 and LNCaP cells on dentin surfaces. (A) Fluorescence images of DAPI-labelled PC3 cells adhering to polished (untreated) dentin (left), acid treated dentin (middle), and collagenase treated dentin for 30 minutes (top) or 60 minutes (bottom) (left panel). For quantification, the numbers of adherent PC3 cells were counted with the cell counter tool in Image J software on all three dentin specimen, respectively. For each dentin specimen, 4 randomly-selected positions on 2 independent dentin chips have been examined. Mean cell numbers and standard errors of cell numbers of adhering cells after 30 minutes (dark grey) and 60 minutes (light grey) are displayed in the bar chart (right panel) (B). Fluorescence images of adhering DAPI-labelled LNCaP cells after 30 minutes (top) or 60 minutes (bottom) on untreated dentin (left), acid treated (middle), and collagenase treated dentin chips (right) (left panel). Quantification of adherent LNCaP cell numbers was performed as for the PC3 cells. A significant p-value from an unpaired t-test of the compared data is marked by *(p<0.05). Corresponding p-values are added under the brackets. All scale bars: 100 μm.

For cell adhesion assays, four randomly selected positions on two independent dentin chips from each dentin type were examined. Mean cell numbers and standard deviations of the numbers of the adhering cells after 30 minutes and 60 minutes were calculated. An unpaired t-test was used to investigate whether the cell numbers on acid treated dentin differ significantly from the cell numbers on untreated and collagenase treated dentin. Corresponding p-values were added under the brackets and a significant p value was marked by *(p<0.05) (Fig 1). In SCFS experiments, adhesion rates and average step numbers were obtained from 8 PC3 and 8 LNCaP cells, respectively. A significant p-value from an unpaired t-test of the compared data is marked by *(p<0.05), corresponding p-values were added under the brackets and the measured mean value of each cell is displayed as a red cross (Fig 3).

## Results

### Adhesion of prostate cancer cells increases on demineralized dentin

Fig 1 shows representative fluorescence microscopy images of DAPI labelled PC3 (Fig 1A) and LNCaP cells (Fig 1B) on untreated dentin substrates serving as model system for intact, well mineralized bone (left), acid treated dentin mimicking demineralized bone as caused by lack of vitamin D (middle), and collagenase treated dentin (right). Cells were incubated on the respective substrates for 30 (top) and 60 minutes (bottom) in cell culture medium, before rinsing the samples with PBS buffer to remove cells, which were not firmly attached. On the right hand side of Fig 1, the corresponding cell densities are quantified (mean values from 4 different areas on 2 independent dentin chips for each substrate, incubation time, and cell type; in total, 96 measurements on 24 independent chips). On untreated dentin, the bone metastasis derived PC3 cells have a cell density of 61 cells/mm^2^ after 30 minutes and 69 cells/mm^2^ after 60 minutes incubation time. On acid treated dentin, the PC3 cell densities increase to 117 cells/mm^2^ after 30 minutes and 140 cells/mm^2^ after 60 minutes. On collagenase treated substrates, however, the number of adherent PC3 cells is reduced to 23 cells/mm^2^ and 48 cells/mm^2^ after 30 and 60 minutes, respectively. The lymph node-derived LNCaP cells, on the other hand show much lower cell densities on all three substrates, with 3 and 17 cells/mm^2^ on untreated dentin, 12 and 36 cells/mm^2^ on acid treated and 9 and 24 cells/mm^2^ on collagenase treated dentin.

### Increased surface roughness is not the cause of increase in cell adhesion after acid treatment

To assess the effect of acid and collagenase treatment on the surface properties, AFM was used to image the different dentin substrate topographies (cf. S1 Fig). The surface of untreated dentin is largely characterized by hydroxyapatite, as indicated by blue arrows (S1A Fig). Only in scarce isolated spots, collagen fibrils, which can be identified by their periodic D band structure, are observed. Upon treatment with hydrochloric acid, more collagen fibrils become exposed and cover almost the whole area, as indicated by red arrows (S1B Fig). Subsequent collagenase treatment removes most of the superficial, exposed collagen, and only few fibrils remain detectable. In addition, the surface roughness of the dentin substrates changes considerably due to the different treatments: In AFM images (5x5μm), the lowest roughness is exhibited by the freshly polished untreated dentin chips with values for Ra around 40 nm. This roughness remains about the same for acid treated dentin specimen. Upon the subsequent collagenase treatment, the values increase almost 4-fold to almost 160 nm, most likely due to the removal of accessible collagen from the substrate surface (cf. S2 Fig). Because the adhesion levels of PC3 cells to collagenase treated dentin are reduced even below those measured on the smooth surfaces of untreated dentin, the variation in surface roughness does not explain the observed increase of PC3 adhesion on acid treated dentin. As shown above, for LNCaP cells only a slight increase in adhesion rates is observed on both acid treated and collagenase treated dentin. However, compared to PC3, the LNCaP adhesion rates remain low on all three dentin substrates.

### Dentin demineralization enhances the initial adhesion rate and changes the adhesion type of PC3 cells

**Fig 2:**
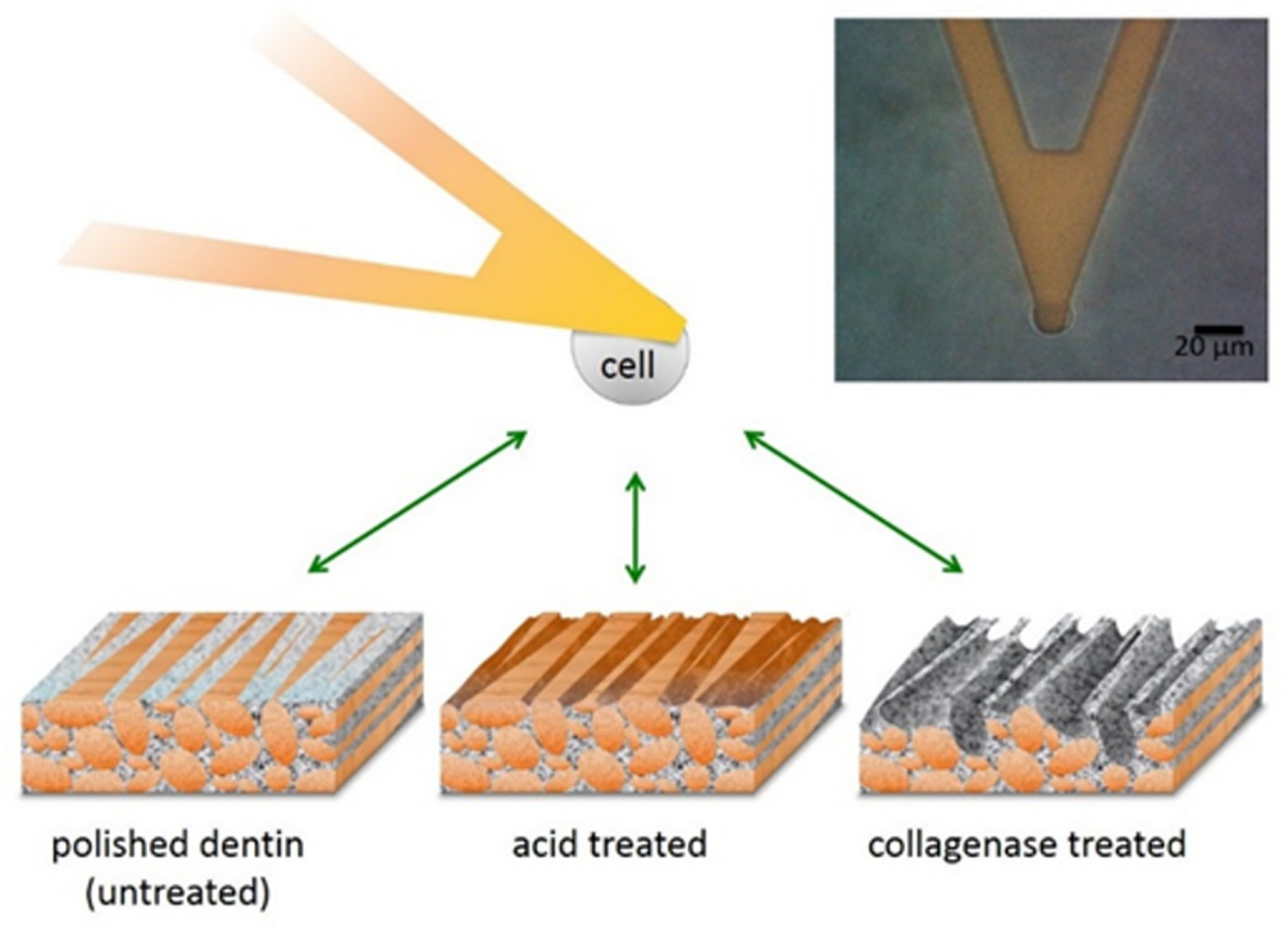
Schematic illustration of the AFM- setup. For AFM-based single cell force spectroscopy, a single prostate cancer cell (either PC3 or LNCaP) was attached to a tipless AFM cantilever and the interaction forces to each of the three different substrates (untreated, acid treated, and collagenase treated dentin) were investigated (not drawn to scale; collagen fibrils depicted in orange, mineral phase in grey). Inserted on the right is a bright field image of a PC3 cell immobilized on the cantilever, before approaching the dentin substrates. (Scale bar: 20 μm).

For cancer cells circulating in the blood stream, the ability to firmly attach to the tissue surface upon initial contact is essential in order to colonize the respective tissue [32–34]. To quantify this initial cell adhesion for PC3 and LNCaP cells, we also conducted AFM-based SFCS, as depicted schematically in Fig 2. Cells from both prostate cancer cell lines, were attached to a tipless AFM cantilever and brought into contact with the three different dentin substrates, mimicking different conditions of the bone microenvironment, and retracted immediately after contact (effective contact time 0.2 s). The insert on the top right side of Fig 2 shows a bright field microscopy image of a PC3 cell attached to an AFM cantilever.

**Fig 3:**
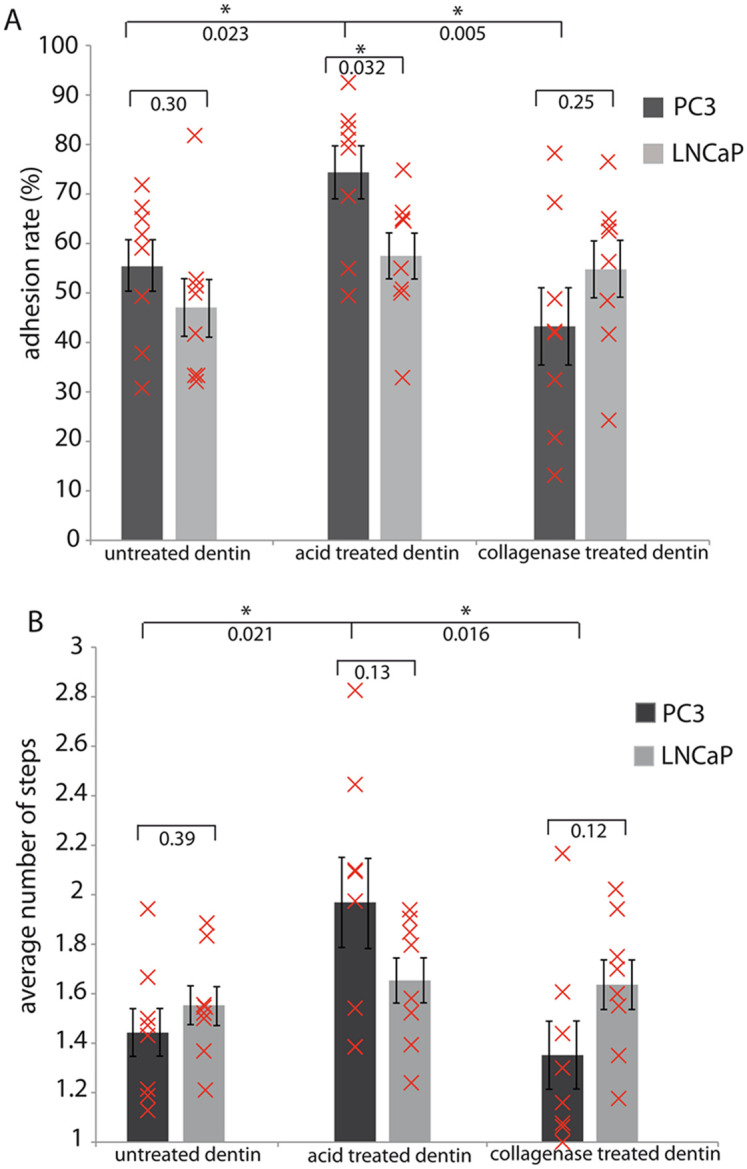
Cell adhesion data derived from single cell force spectroscopy of prostate cancer cells to dentin substrates. (A) The adhesion rate (percentage of force curves with at least one de-adhesion event) and (B) average step number (the average number of de-adhesion events within a successful force curve) of PC3 (dark grey) and LNCaP (light grey) cells on polished (untreated), demineralized and collagenase-treated dentin surfaces. Adhesion rates and average step numbers were obtained from 8 PC3 and 8 LNCaP cells, respectively. Each cell was probed against all three dentin specimen, as depicted in Fig 2 and described in the materials and methods. Error bars correspond to standard error of the mean. A significant p-value from an unpaired t-test of the compared data is marked by *(p<0.05). Corresponding p-values are added under the brackets. The measured mean value of each cell is displayed as a red cross.

**Fig 4:**
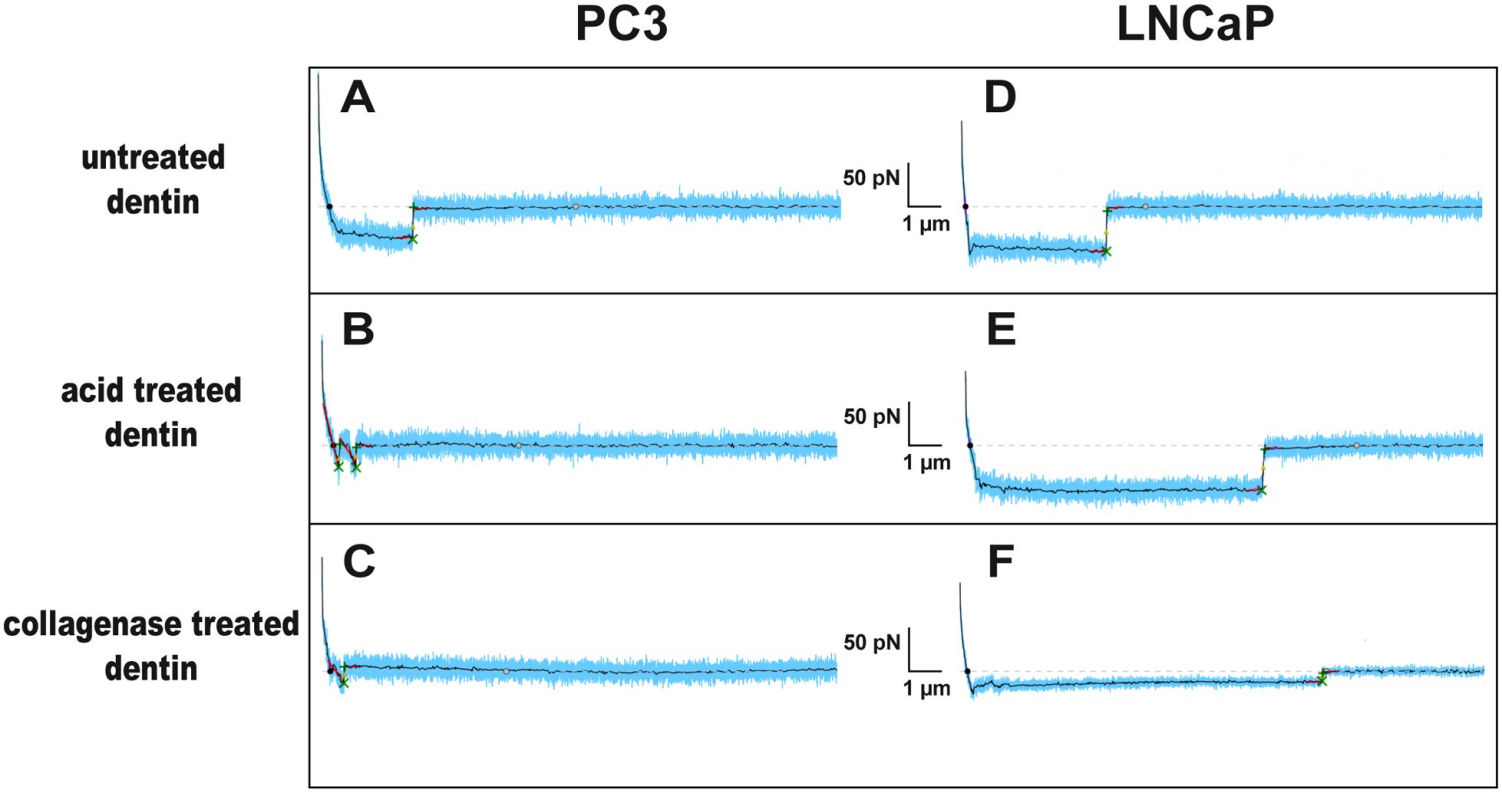
Representative force-distance curves acquired from SCFS experiments. (A) PC3 cells interacting with polished (untreated) dentin, predominantly display 2–4 μm long plateau-like rupture events (tethers). (B-C) After contact to demineralized and collagenase-treated dentin surfaces, PC3 display almost exclusively jump-like ruptures. LNCaP cells display predominately tethers on all three substrates: (D) LNCaP on polished (untreated) dentin, (E) LNCaP on demineralized dentin, (F) LNCaP on collagenase-treated dentin surface. Raw data of force-distant curves is displayed in blue, smoothed curves are displayed in black. Ruptures are indicated by green crosses, slopes before and after ruptures are colored in red. Slopes < -10 pN/nm correspond to jump-like ruptures, other slopes (⁓ 0 pN/nm) correspond to tethers.

Fig 3 summarizes the adhesion rates and the number of steps observed in the SCFS experiments for the investigated cancer cell lines. The adhesion rates were calculated as the fraction of force-distance curves with at least one cell detachment event and are thus a measure for the likelihood of a cell-substrate interaction [10] (exemplary force-distance curves are given in Fig 4 and will be discussed in more detail in the next paragraph). Fig 3A shows the adhesion rates for all three dentin substrates. For PC3 cells, the adhesion rate significantly increased from 55% on untreated dentin to 74% on acid treated dentin. On collagenase treated substrates, the adhesion rate of PC3 cells significantly decreased to only 43%. For LNCaP cells on the other hand, the increase of the adhesion rate on acid treated dentin was not statistically significant. The adhesion rate of LNCaP cells on the collagenase treated samples is almost the same as on the acid treated samples. In addition, we have analyzed the average number of detachment steps per curve. This parameter summarizes the number of individual adhesion sites observed in force curves where a cell-substrate interaction occurred [10] and correlates with the density of binding sites between cell and substrate at the contact site. This step number (Fig 3B) shows a similar picture: for PC3 cells the average number of steps increases from 1.44 on untreated dentin to 1.97 on acid treated dentin, and drops to 1.35 on collagenase treated dentin. With 1.55, 1.65, and 1.64 on untreated, acid treated and collagenase treated dentin, respectively, no significant differences in average step numbers are observed for LNCaP.

**Fig 5:**
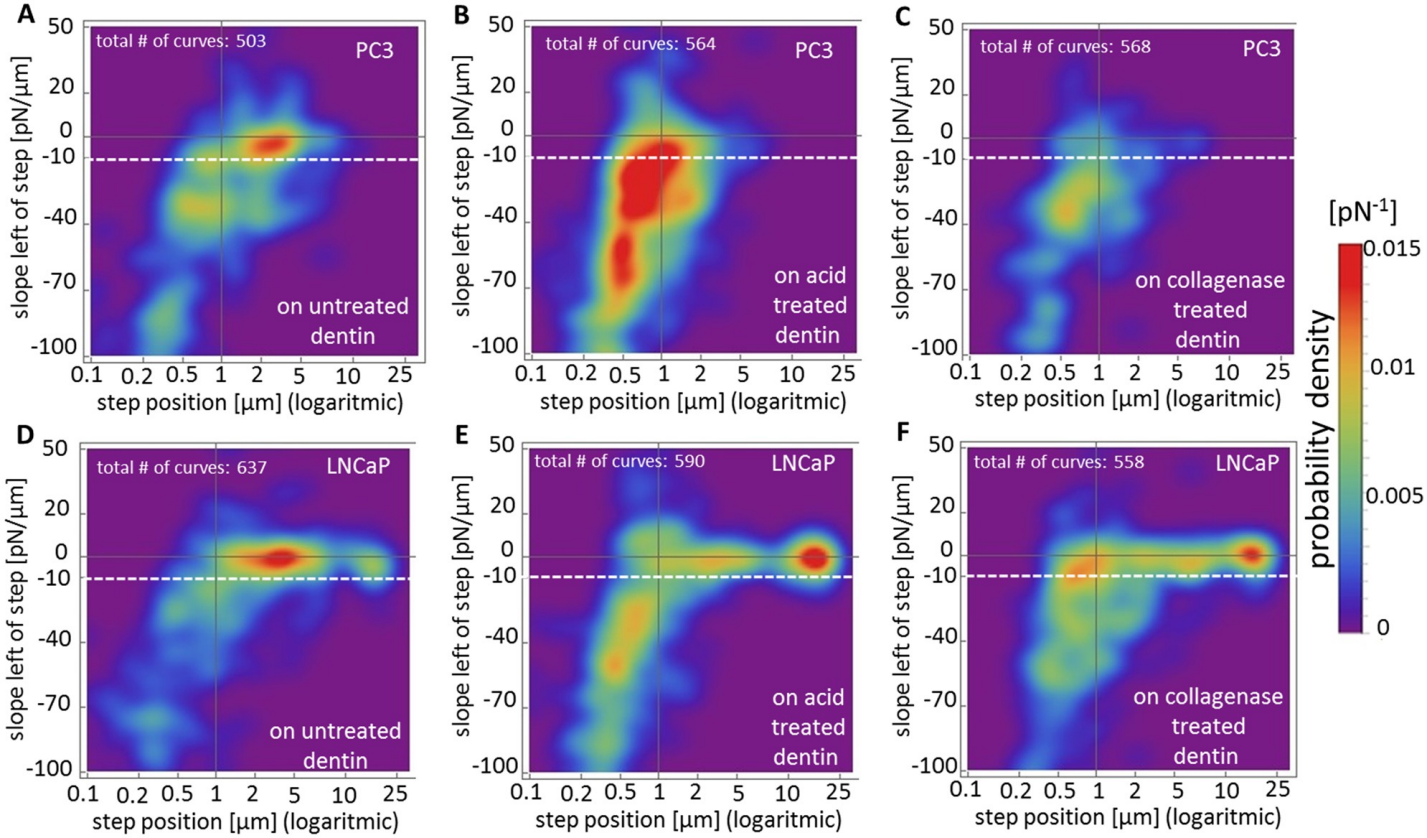
Two-dimensional probability density plots of prostate cancer cell interaction with dentin surfaces. The plot displays the step position versus the slope of all rupture events observed for PC3 cells on untreated (A), acid treated (B) and collagenase-treated dentin (C), and LNCaP cells on untreated (D), acid treated (E) and collagenase treated dentin (F). The color code represents the probability density of a rupture event occurring at the designated slope and position. The distributions were normalized with respect to the total number of force curves performed on each specimen per cell type.

Interestingly, for the PC3 cells not only the adhesion rate and number of steps increase considerably on acid treated dentin, compared to untreated dentin, but also the type of interaction changes. On untreated dentin, force curves of PC3 cells show mostly long smooth plateaus ending in discrete force steps, indicating a rupture event, as depicted in Fig 4A. Such plateaus have been attributed to lipid tethers being pulled out of the plasma membrane [35, 36]. On acid treated dentin, however, the force curves of PC3 cells reveal mostly (but note exclusively) short jump-like rupture events, with a steep decrease in force (slopes well below -10pN) immediately before the discrete step indicating the rupture event (Fig 4B). Such ruptures are characteristic for interactions mediated *via* cytoskeleton linked membrane receptors, as has been shown in a previous study [31]. On collagenase treated dentin, force curves of PC3 cells still display a few of these short jump-like steps (Fig 4C), but the total number of interactions is considerably reduced, as can be seen in Fig 5C and will be discussed in the next paragraph. Force curves of LNCaP cells, on the other hand, show mostly long smooth plateaus on all three substrates (Fig 4D–4F), with extended plateau lengths on acid and on collagenase treated dentin chips (Fig 4E and 4F).

Fig 5 summarizes step positions and slopes immediately before the rupture events of all force curves recorded in a color-coded two-dimensional plot visualizing the probability density (rupture probability per unit area of the plot [pN^-1^]) of jump-like rupture events (slopes below -10 pN/μm) versus tethers (slope between 0 and -10 pN/μm). As explained above, these jump-like rupture events have been attributed to cytoskeleton linked transmembrane receptors [31], while tethers consist of membrane tubes which are pulled out of the plasma membrane [35, 36].

For PC3 cells on untreated dentin (Fig 5A), the distribution exhibits a global maximum in the tether region (above the white dashed line) at step positions between 2 and 4 μm and slopes between 0 and -10 pN/μm (red area), as well as a much smaller, local maximum at step positions between 0.5 and 1 μm and slopes between -30 and -40 pN/μm (yellow), characteristic for jumps. On acid treated dentin (Fig 5B), the overall number of adhesion events increases considerably, as indicated by the large red and yellow areas, where red encodes for the highest probability and thus the highest overall number of rupture events. Here, the former maximum in the membrane tether region at long rupture lengths disappears, and much shorter ruptures (< 1 μm) with slopes approximately between -10 and -70 pN/μm (jumps) are predominant, indicated by the large red areas. On collagenase treated dentin (Fig 5C), the total number of rupture events for PC3 cells is much lower (mainly blue and green areas), but shows a similar distribution of rupture lengths and slopes as on acid treated specimen: The maximum of interactions can still be observed in the jump region (yellow area) with short steps at around 0,5 μm and at slopes between -15 and -40 pN/ μm, while rupture events in the tether region have declined drastically.

LNCaP cells on the other hand show predominantly long tether-like force curves on all three substrates (Fig 5D–5F, red areas). Interestingly, on both, the acid treated and collagenase treated dentin, the global maximum is shifted to very high step positions around 20 μm for LNCaP (Fig 5E and 5F). Tether ruptures at these step positions can be found on untreated dentin samples as well, but they have a much lower probability than those at step positions between 2 and 5 μm. Additionally, for LNCaP cells, only few jump-like ruptures with slopes between -15 and -70 pN/μm and step positions up to 0.5 μm can be observed on acid treated substrates. Collagenase treatment diminishes this type of cytoskeleton- linked interaction.

## Discussion

For the bone invasive PC3 cells, the fluorescence microscopy-based adhesion assay reveals an almost two-fold increase of cell densities on acid treated dentin, compared to untreated dentin both after 30 and after 60 minutes incubation time. On surfaces treated with collagenase, the cell densities drop even below the levels observed on untreated samples. Depending on the preparatory treatment, the analyzed dentin chips differ considerably with respect to their surface properties. The removal of hydroxyapatite through acid treatment does not affect the surface roughness dramatically, as roughness values calculated from AFM images (5x5 μm^2^) almost remain the same. (cf. S1 Fig). Only after subsequent collagenase treatment, an almost 4-fold increase of the surface roughness compared to the values obtained from untreated surfaces, between approximately 40 nm– 160 nm is observed. This ⁓120 nm increase in surface roughness is comparable to the diameter of the collagen fibrils in dentin of ⁓ 100–120 nm [37]. However, despite of the larger roughness on collagenase treated dentin compared to the other surfaces, the PC3 cell densities obtained from the classical adhesion assay and the adhesion rates obtained from SCFS on collagenase treated dentin remain well below the cell densities and adhesion rates found on both of the other substrates. The conclusion to be drawn is that surface roughness, which may be a cause of enhanced cell adhesion [26, 38, 39], can be ruled out as explanation for the observed increase in PC3 cell adhesion on acid treated dentin.

AFM images reveal that the tested dentin specimen differ with respect to their collagen exposure at the surface: On untreated chips, almost the entire surface is covered with hydroxyapatite minerals. In this native state, the individual mineralized collagen fibrils are surrounded with hydroxyapatide crystals [23, 40]. Acid treatment removes the hydroxyapatite from the surface and uncovers the collagen fibrils. Grünherz et al. could show that acid treatment generates a demineralized seam on the dentin surface that closely resembles the osteoid seam found in bone biopsies from osteomalacia patients [22]. Collagenase treatment removes almost all superficial collagen fibrils. We conclude that accessible collagen fibrils on the substrate surface play a key role in the adhesion of PC3 cells to dentin. The lymph node derived LNCaP cells, on the other hand, show almost one order of magnitude lower overall adhesion to all three substrates in the fluorescence microscopy images, than the bone derived PC3 cells. They exhibit slightly higher cell densities on both acid treated and collagenase treated dentin than on untreated dentin, suggesting that LNCaP adhesion to dentin is not collagen specific.

In contrast to the fluorescence-based adhesion assay, where the number of firmly attached cells after 30 and 60 minutes was analyzed, our SCFS experiments aim at gaining information on initial cell adhesion occurring within the first 200 ms of contact and at identifying individual attachment sites that might act as initial cell-substrate contacts. It should be emphasized, that the information on the initial adhesion events gained by SCFS is complementary, but not identical to the information on longer-term adhesion, gained from the fluorescence microscopy experiments. In the SCFS experiments, both the adhesion rate and number of detachment steps of PC3 cells on acid treated dentin increase again significantly, compared to untreated dentin. The adhesion rate as well as the number of detachment steps on collagenase treated dentin drop to values corresponding to those observed on untreated dentin. These findings emphasize the importance of collagen for PC3 cell adhesion, which goes in line with a previous study where 66% adhesion rate was observed in SCFC experiments when probing PC3 cells on collagen-I coated glass slides, whereas controls with PC3 on BSA coated slides yielded 35% adhesion rate [31]. The same magnitude was reported for PC3 cells treated with a monoclonal antibody against CD29/ integrin β1 for blocking this receptor [31]. Taken together, these observations suggest that in the AFM experiments PC3 adhesion is mainly collagen modulated.

Conversely, and in agreement with our fluorescence-based assay, adhesion of LNCaP does not depend on collagen exposure, since neither the adhesion rate nor the number of detachment steps change significantly when probing the different dentin specimen. Unlike in the fluorescence-based assay, where LNCaP cells show substantially lower adhesion than PC3 cells on all three substrates, in the AFM experiments, the adhesion rates and step numbers for LNCaP cells are comparable to those of PC3. LNCaP adhesion increases slightly on acid treated dentin and remains the same on collagenase treated samples. The increased adhesion of LNCaP relative to PC3 in the AFM experiments, compared to the fluorescence microscopy, may in part be caused by differences in cell stiffness between the two cell lines. The elastic modulus of PC3 cells exceeds that of LnCAP cells by the factor 1.5–2.2 [11, 41]. This leads to an increase in the contact area of LnCAP cells by the factor 1.4–1.7 compared to PC3, when the cells are pushed against the substrate surface with a contact force of approximately 100 pN in the AFM set up [42] (cf. S1 Table). In contrast, in the fluorescence-based adhesion assay, the cells are suspended freely above the substrate surface and the initial contact area is small for both cell types. The larger surface area of cells, which are pushed against the substrate allows a larger number of possible interactions between cell and substrate [43] and may therefore explain, why in AFM experiments the collagen independent LNCaP adhesion rates are in about the same order of a magnitude as the values for PC3 adhesion, while in the fluorescence-based adhesion assay, the density of adherent LNCaP cells is explicitly lower. These observations are corroborated by fact that, despite of the much lower adhesion of LNCaP in the fluorescence assay, in the SCFS experiments, the mean detachment forces of both cell lines are nearly identical on each of the three dentin substrates (S3 Fig). Furthermore, this is also in accordance with findings by Yu et al. that the initial adhesion of PC3 and LNCaP to collagen is comparable in SCFS experiments and that PC3 adhesion strengthens over time, while LNCaP adhesion remains nearly constant [44], which could be yet another reason for the significantly higher adhesion rates of PC3 compared to LNCaP after 30 and 60 minutes in the fluorescence based assay.

Interestingly, in the force curves derived from PC3 cells on untreated dentin, long plateaus, which are characteristic for lipid tethers being pulled out of the cell membrane, represent the majority of the interactions. Shorter jump-like rupture events, caused by cytoskeleton-linked transmembrane receptors (short jumps) can be identified as well, but to a smaller extent [31]. On acid treated dentin, however, PC3 adhesion increases and PC3 show predominantly (but not entirely) short jump-like steps. This observation coincides with a recent study, where the major interaction type of PC3 cells on collagen (Col-1) coated glass slides was clearly identified as jump-like. This study also showed that the PC3 binding to collagen can be effectively blocked and reduced to the level of non-specific binding by incubating the cells with an anti-β1 antibody [31]. This supports the hypothesis that collagen specific transmembrane receptors, such as α1β1, and α2β1 integrin may be responsible for the cell-surface interaction on the demineralized surface. Both collagen-binding integrins (α1β1, and α2β1) are strongly expressed in PC3 cells but only barely detectable in LNCaP [10]. The jump-like events remain the dominant type of interaction for PC3 cells also on collagenase treated samples, where the total number of interactions is however, significantly reduced. These few remaining jump-like adhesion events are most likely the consequence of few remaining collagen fibrils, which were not degraded by collagenase treatment. In addition, PC3 cells present other RGD-binding integrins on their surface, such as α_v_β_3_ and α_5_β_1_ [45], which bind the dentin matrix protein DMP1 [46] as well as fibronectin [47], which is present in high amounts in the dentin matrix [48]. These integrin receptors may therefore also contribute to the jump-like interactions on collagenase treated dentin. However, it should be emphasized again, that although the relative number of jumps (vs. tethers) remains high for PC3 cells on collagenase treated dentin, the absolute number of interactions is reduced to a level below the level observed on untreated dentin, as indicated by the much lower probability densities in Fig 5C compared to Fig 5B and the reduced overall adhesion rates and step numbers in Fig 3.

LNCaP cells, on the other hand, behave again quite differently on these three substrates: In the SCFS experiments, they show mostly plateaus on all three substrates, indicating that their adhesion to dentin is not predominantly mediated by cytoskeleton-linked receptors. Only few jump-like ruptures appear on acid treated dentin and even less on collagenase treated chips. Quantitative real-time-PCR revealed that in LNCaP the expression of α1, α2, and β1 integrin is detectable, although the expression levels are significantly lower than in PC3 [10]. Most probably, these collagen-binding integrins are therefore responsible for the rarely occurring jumps on untreated and collagenase treated dentin samples, revealed by the probability plots (Fig 5A–5C). However, the dominating tethers can be grouped on basis of their step position: Short tethers with step positions from 2 to 5 μm represent the main type of interaction on untreated dentin samples. Interestingly, these short tethers also characterize the interaction of PC3 cells to the same substrate, which suggests a common type of interaction on the hydroxyapatite surface for both cell types. In contrast, the majority of tethers on acid treated and collagenase treated dentin formed by LNCaP cells has a length of about 20 μm. For PC3 cells, such long tethers were neither observed in adhesion experiments on dentin nor on other substrates like Col-I-coated or BSA-coated glass slides [10, 31]. The long tethers of LNCaP indicate that these cells might possess a larger membrane reservoir than PC3 cells, which is in line with the smaller value for the Young’s modulus [49, 50]. We hypothesize that pulling out of these long tethers on acid treated and collagenase treated dentin is mediated either by the interaction of collagen with an alternative, non-integrin receptor which is not linked to the cytoskeleton or by the interaction of a non-collagenous protein (NCP) from the dentin matrix with a not yet identified receptor on the LNCaP surface. Nevertheless, the exact reason for this long tether formation has to be clarified in a subsequent study.

## Conclusion

In the present study, we have shown that the demineralization of dentin, enhances cell adhesion of the bone invasive prostate cancer cell line PC3. Our data suggests that accessible collagen fibrils at the substrate surface play a key role in this process, although other ECM proteins may also contribute. Dentin was chosen because it is a bone like biomimetic surface, suitable for fluorescence and AFM based cell adhesion assays. Attachment of PC3 to a poorly mineralized bone surface via interaction with collagen may thus be a first step in bone colonization of PC3. Our results may therefore contribute to a better understanding of bone colonization by cancer cells and thereby help to develop better and more effective therapeutic strategies. In particular, they may aid to gain a more complete picture of the role of vitamin D and bone mineralization in cancer treatment.

## Supporting information

S1 Fig AFM deflection images of the dentin microstructure. (A) After polishing, the dentin surface is smooth and predominantly covered with hydroxyapatite crystals, visible as roundish an overlapping particles in the nanometer scale. This dentin surface corresponds to the untreated and well mineralized dentin in the results part. (B) Treatment of polished dentin with 1 M hydrochloric acid for 5 minutes removes the hydroxyapatite and the collagen fibrils, identified by their periodic D-band structure are exposed. This surface is referred to as acid treated dentin. (C) By incubation of the dentin in collagenase solution overnight, the exposed collagen is removed and the dentin surface is mainly characterized by hydroxyapatite again. Corresponding height images (not shown) reveal a marked increase in surface roughness. This is the collagenase treated dentin surface. (D) 1μm x 1μm close up from (B, white square) showing the 67 nm D-band structure of collagen. The inserted graph shows the topography of the highlighted collagen profile in (D) which reveals the periodic D-band structure of collagen with periodic repeats at approximately every 67 nm. All images were acquired in contact mode in air at 1 Hz scan rate using an MLCT AFM cantilever with pyramidal tip and a nominal spring constant of 10 mN/m. (Scale bars in A, B and C = 2μm, in D = 0,4 μm).
(TIF)

S2 Fig Root Mean Square (Rq) and Average Roughness (Ra) of the various dentin specimen as determined from AFM topography images. Two AFM images (5 μm x 5 μm, height images) of each dentin specimen (untreated, acid treated and acid + collagenase treated dentin) were analyzed as follows: Rq and Ra values of 4 areas (2 μm x 2 μm) from each image were calculated, using the JPK Data Processing software. The bars show the mean roughness values of in total 8 areas per dentin specimen (error bars correspond to standard deviation). A significant p-value from an unpaired t-test of roughness data of collagenase treated sample with respect to untreated dentin sample is marked by *(p<0.01). The mean roughness (Rq) for untreated dentin chips is 53 nm ± 23 nm, for acid treated dentin 44 nm ± 6 nm and for acid and collagenase treated dentin surfaces 196 nm ± 44 nm. Images were acquired in tapping mode in PBS solution at 0.9 Hz scan rate using an MLCT AFM cantilever with pyramidal tip and a nominal spring constant of 10 mN/m.
(TIF)

S3 Fig Detachment forces of PC3 and LnCAP cells. Detachment force of PC3 (dark grey) and LnCAP (light grey) cells on untreated, well-mineralized dentin (left), acid treated dentin (middle) and collagenase treated dentin (right). Detachment forces were obtained from 8 PC3 and 8 LNCaP cells, respectively. Each cell was probed against all three dentin specimen. The columns show the mean value, the error bars correspond to standard deviation and the red crosses represent the mean detachment force of each individual cell.
(TIF)

S1 Table Calculation of contact area in SCFS [51]. (DOCX)
