# Supplementary figures and images for: Inadequate tissue mineralization promotes cancer cell attachment
Source: PLoS One. 2020 Aug 28;15(8):e0237116. doi: 10.1371/journal.pone.0237116 (PMC7454967; doi:10.1371/journal.pone.0237116)

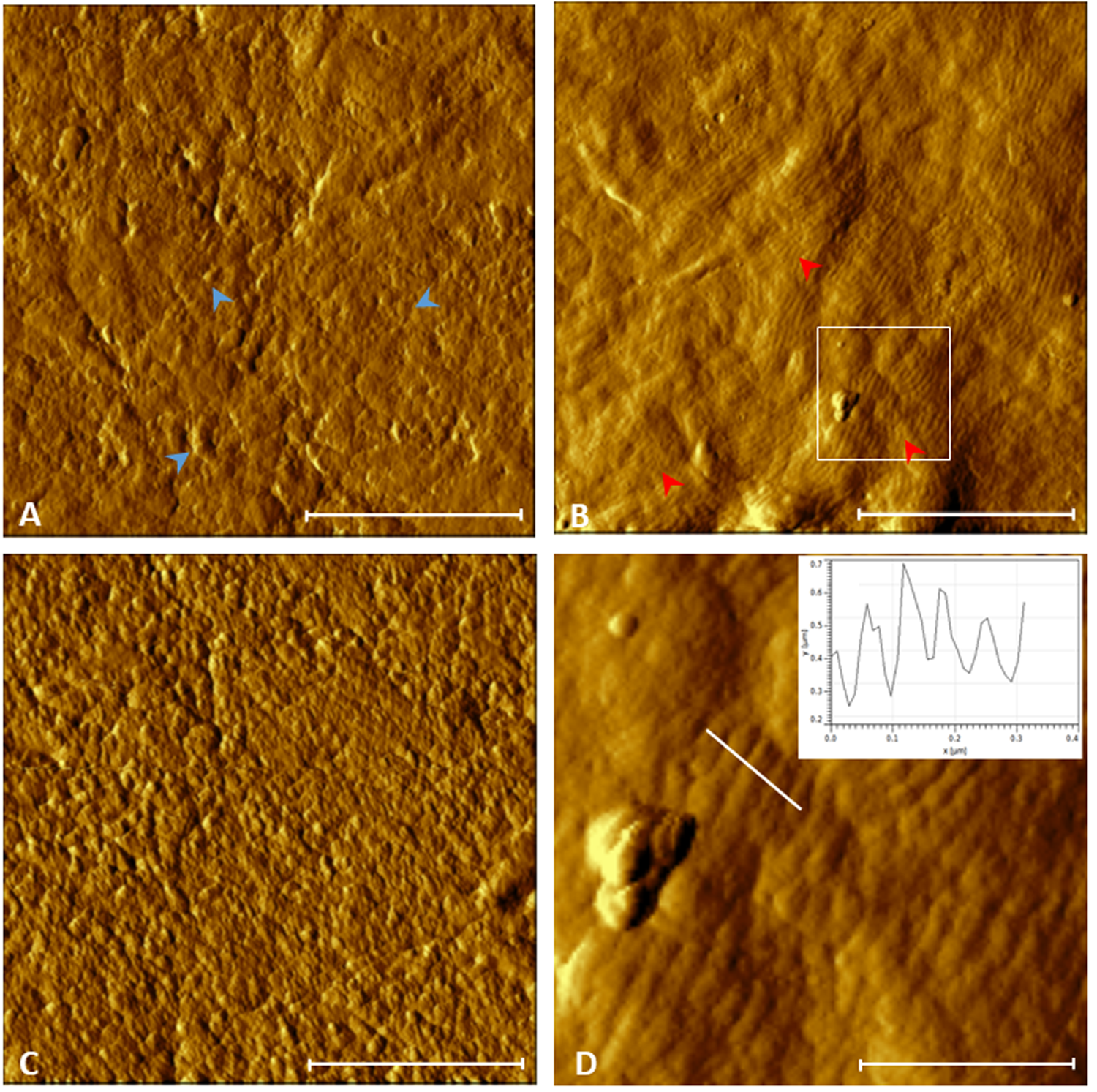

Supplement: S1 Fig — (A) After polishing, the dentin surface is smooth and predominantly covered with hydroxyapatite crystals, visible as roundish an overlapping particles in the nanometer scale. This dentin surface corresponds to the untreated and well mineralized dentin in the results part. (B) Treatment of polished dentin with 1 M hydrochloric acid for 5 minutes removes the hydroxyapatite and the collagen fibrils, identified by their periodic D-band structure are exposed. This surface is referred to as acid treated dentin. (C) By incubation of the dentin in collagenase solution overnight, the exposed collagen is removed and the dentin surface is mainly characterized by hydroxyapatite again. Corresponding height images (not shown) reveal a marked increase in surface roughness. This is the collagenase treated dentin surface. (D) 1μm x 1μm close up from (B, white square) showing the 67 nm D-band structure of collagen. The inserted graph shows the topography of the highlighted collagen profile in (D) which reveals the periodic D-band structure of collagen with periodic repeats at approximately every 67 nm. All images were acquired in contact mode in air at 1 Hz scan rate using an MLCT AFM cantilever with pyramidal tip and a nominal spring constant of 10 mN/m. (Scale bars in A, B and C = 2μm, in D = 0,4 μm). (TIF) [file pone.0237116.s001.tif]

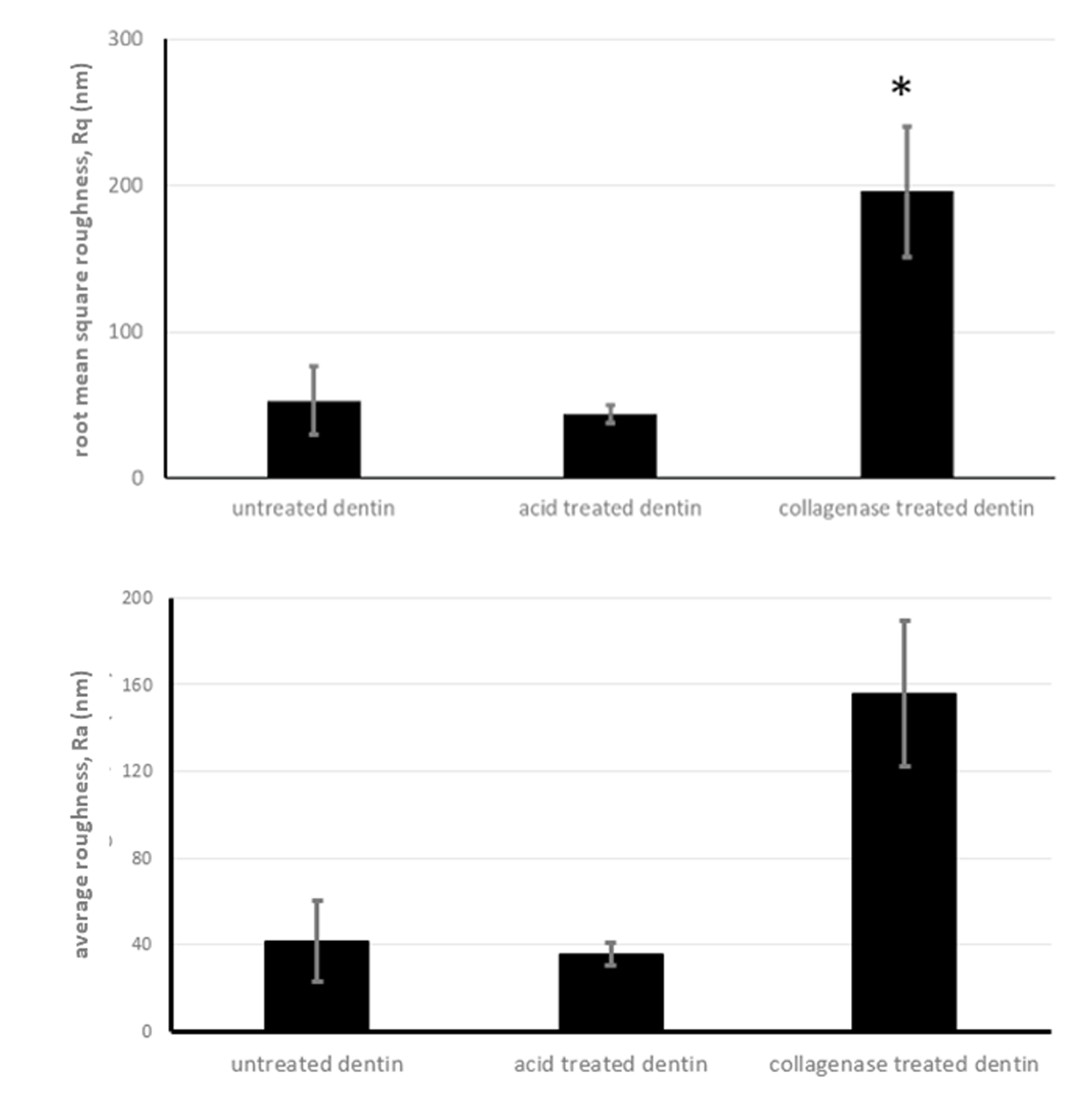

Supplement: S2 Fig — Two AFM images (5 μm x 5 μm, height images) of each dentin specimen (untreated, acid treated and acid + collagenase treated dentin) were analyzed as follows: Rq and Ra values of 4 areas (2 μm x 2 μm) from each image were calculated, using the JPK Data Processing software. The bars show the mean roughness values of in total 8 areas per dentin specimen (error bars correspond to standard deviation). A significant p-value from an unpaired t-test of roughness data of collagenase treated sample with respect to untreated dentin sample is marked by *(p<0.01). The mean roughness (Rq) for untreated dentin chips is 53 nm ± 23 nm, for acid treated dentin 44 nm ± 6 nm and for acid and collagenase treated dentin surfaces 196 nm ± 44 nm. Images were acquired in tapping mode in PBS solution at 0.9 Hz scan rate using an MLCT AFM cantilever with pyramidal tip and a nominal spring constant of 10 mN/m. (TIF) [file pone.0237116.s002.tif]

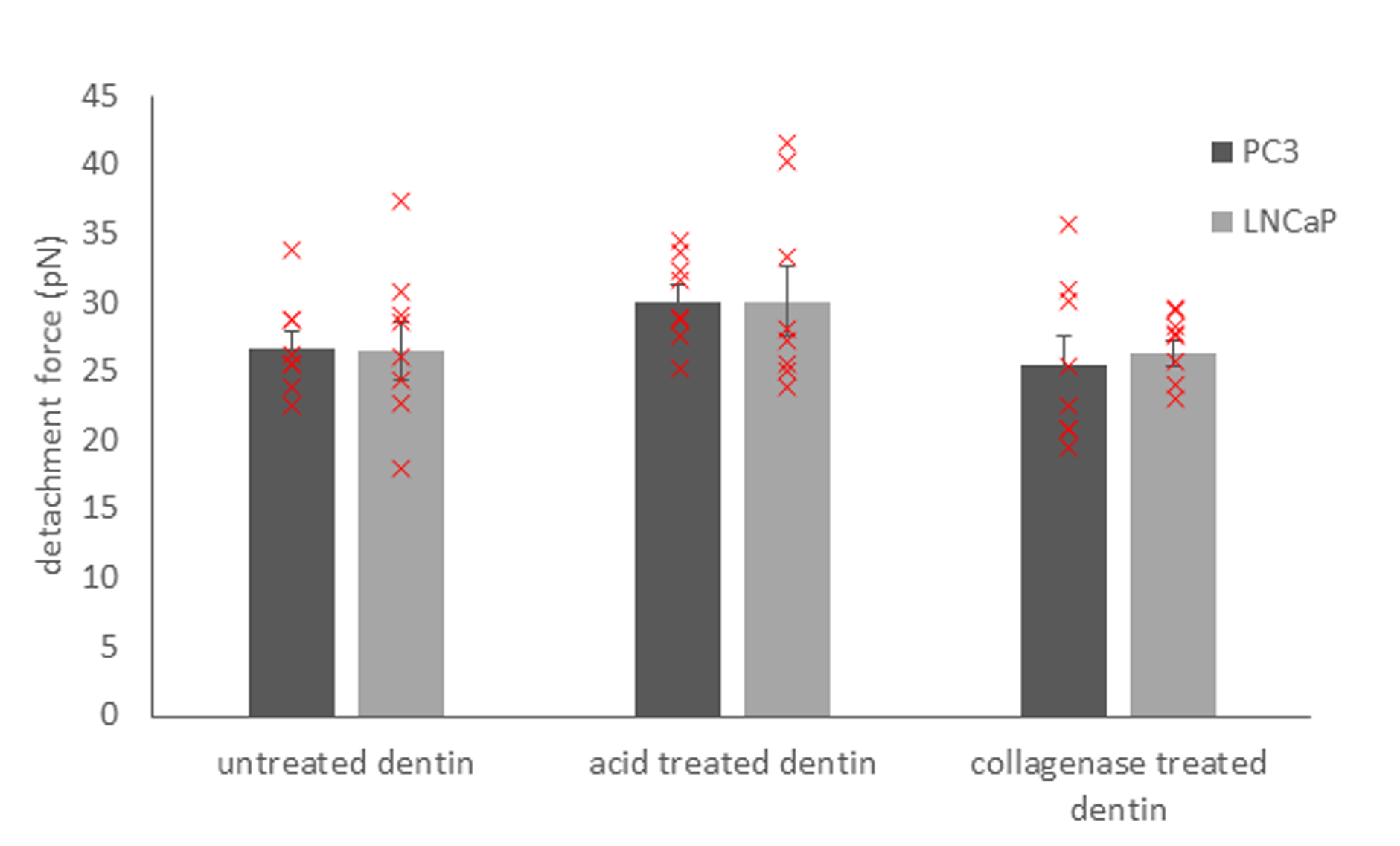

Supplement: S3 Fig — Detachment force of PC3 (dark grey) and LnCAP (light grey) cells on untreated, well-mineralized dentin (left), acid treated dentin (middle) and collagenase treated dentin (right). Detachment forces were obtained from 8 PC3 and 8 LNCaP cells, respectively. Each cell was probed against all three dentin specimen. The columns show the mean value, the error bars correspond to standard deviation and the red crosses represent the mean detachment force of each individual cell. (TIF) [file pone.0237116.s003.tif]
