# Supplementary material for: Inadequate tissue mineralization promotes cancer cell attachment
Source: PLoS One. 2020 Aug 28;15(8):e0237116. doi: 10.1371/journal.pone.0237116 (PMC7454967; doi:10.1371/journal.pone.0237116)
Supplement: S1 Table — (DOCX) [file pone.0237116.s004.docx]

**S1 Table. Calculation of contact area in SCFS.**

| **Cell Type** | **Data Source** | **Young’s modulus (YM)**  **/ Elastic modulus (EM)**  **[kPa]** | **Calculated contact area [µm^2^]** | **Contact area ratio LnCAP compared to PC3** |
| --- | --- | --- | --- | --- |
| **LnCAP** | Docheva et al, 2010 | >2 | **1.35** | **1.77** |
|  | Liu et al, 2019 AFM data | 1.61 | **1.56** | **1.35** |
|  | Liu et al, 2019, microfluidic data | 1.08 | **2.03** | **1.44 – 1.70** |
| **PC3** | Docheva et al, 2010 | 4.7 | **0.76** |  |
|  | Liu et al, 2019 AFM data | 2.53 | **1.15** |  |
|  | Liu et al, 2019, microfluidic data | 1.87 (lowest value) | **1.41** |  |
|  | Liu et al, 2019, microfluidic data | 2.40 (highest value) | **1.19** |  |

Contact area of PC3 and LnCAP cells was calculated on basis of Young’s modulus values of both cell lines derived from earlier AFM measurements [[51](#_ENREF_51)] and on Young’s modulus and Elastic modulus values determined by Liu et al in 2019 through AFM measurements and microfluidic experiments [[41](#_ENREF_41)], respectively. The contact area was calculated as follows:

$A=\pi\left[ \frac{F\cdot R\cdot\left( 1-\nu^{2} \right)}{E} \right]^{\frac{2}{3}}$,

where *A* is the contact area, *F* the contact force (100 pN), *R* the cell radius (10 µm), $\nu$ the poison ratio (0.5) and *E* the Young’s modulus. [[51](#_ENREF_52), [52](#_ENREF_53)]. In our SCFS experiments, the LnCAP cells have a 1.4 – 1.7 fold larger contact area.

**References supporting information**

**[41] Liu N, Du P, Xiao X, Liu Y, Peng Y, Yang C and Yue T 2019 Microfluidic-Based Mechanical Phenotyping of Androgen-Sensitive and Non-sensitive Prostate Cancer Cells Lines *Micromachines* 10 602**

[51] Hertz H R 1882 *Gesammelte Werke (Collected Works)*, (Leipzig, Germany)

[52] Wikipedia 2020 June 26 Cell mechanics. (Wikipedia)
